# Curcumin and Curcuma longa Extract in the Treatment of 10 Types of Autoimmune Diseases: A Systematic Review and Meta-Analysis of 31 Randomized Controlled Trials

**DOI:** 10.3389/fimmu.2022.896476

**Published:** 2022-08-01

**Authors:** Liuting Zeng, Tiejun Yang, Kailin Yang, Ganpeng Yu, Jun Li, Wang Xiang, Hua Chen

**Affiliations:** ^1^ Department of Rheumatology and Clinical Immunology, Peking Union Medical College Hospital, National Clinical Research Center for Dermatologic and Immunologic Diseases (NCRC-DID), Key Laboratory of Rheumatology and Clinical Immunology, Ministry of Education, Chinese Academy of Medical Sciences & Peking Union Medical College, Beijing, China; ^2^ Department of Orthopedics, People’s Hospital of Ningxiang City, Ningxiang, Hunan, China; ^3^ Key Laboratory of Hunan Province for Integrated Traditional Chinese and Western Medicine on Prevention and Treatment of Cardio-Cerebral Diseases, Hunan University of Chinese Medicine, Changsha, Hunan, China; ^4^ Department of Rheumatology, The First people’s Hospital Changde City, Changde, Hunan, China

**Keywords:** curcumina, curcuma longa extract, autoimmune disease, rheumatoid arthritis, systemic lupus erythematosus, psoriasis, systematic review

## Abstract

**Objective:**

To evaluate the randomized controlled trials (RCTs) of Curcumin and Curcuma longa Extract in the treatment of autoimmune diseases.

**Methods:**

Databases such as Embase, Web of Science, PubMed and The Cochrane Library were searched from the database establishment to February 2022 to collect RCTs of Curcumin and Curcuma longa Extract in the treatment of autoimmune diseases. Then the literature was screened and the data were extracted. Meta-analysis was performed using RevMan 5.3 software.

**Results:**

A total of 34 records were included, involving 31 RCTs and 10 types of autoimmune disease. Among them, ankylosing spondylitis (AS) involves one RCT, Behcet ‘s disease (BD) involves one RCT, Crohn ‘s disease involves two RCTs, multiple sclerosis (MS) involves two RCTs, oral lichen planus involves six RCTs, psoriasis involves two RCTs, rheumatoid arthritis (RA) involves five RCTs, systemic lupus erythematosus (SLE) involves two RCTs, arteritis involves one RCT, ulcerative colitis (UC) involves nine RCTs. Among them, most of the RCTs of ulcerative colitis (UC), oral lichen planus, RA showed that curcumin and curcumin extracts improved clinical or laboratory results. Crohn ‘ s disease, MS, SLE, psoriasis included two RCTs; they all showed improvements (at least one RCT reported improvements in clinical outcomes). AS, BD and arteritis included only one RCT, and the clinical results showed improvement. However, due to the small number of RCTs and the small number of patients involved in each disease, there is still a need for more high-quality RCTs.

**Conclusion:**

Curcumin and Curcuma longa Extract had good clinical efficacy in the treatment of Psoriasis, UC and RA, so Curcumin and Curcuma longa Extract could be used in the treatment of the above diseases in the future. The results of Meta-analysis showed that Curcumin and Curcuma longa Extract did not show efficacy in the treatment of oral lichen planus, while Takayasu arteritis, SLE, MS, AS, BD and CD did not report sufficient clinical data for meta-analysis. Therefore, large-sample, multi-center clinical trials are still needed for revision or validation.

## 1 Introduction

Autoimmune diseases refer to diseases caused by the damage of the body’s own tissues caused by the immune response of the body to self-antigens (1). The main reason is that the autoimmune tolerance state is broken or the autoimmune cells are abnormally regulated under the induction of some internal and external factors such as genetic factors and environmental factors, and the immune system produces a persistent and protracted immune response to self-antigens (2, 3). Autoimmune diseases generally include systemic lupus erythematosus (SLE), Sjögren’s syndrome, multiple sclerosis (MS), rheumatoid arthritis (RA), Behcet’s disease (BD), giant cell arteritis, Takayasu arteritis, Psoriasis, ankylosing spondylitis (AS), Oral lichen planus and more than 120 diseases (4, 5). Autoimmune diseases can be induced in a variety of ways, and symptoms vary depending on the disease and the organs involved. The presence of autoimmune disease often requires some blood tests to confirm (6). Treatment for autoimmune diseases depends on the type and severity of the condition. Commonly used drugs include non-steroidal anti-inflammatory drugs (NSAIDs), biologically targeted drugs (such as ustekinumab), and traditional immune-suppressing drugs (such as azathioprine and cyclosporine) (7–9). However, these drugs not only suppress the autoimmune response, but also inhibit the body’s ability to defend itself against foreign invasion, including infection-causing microorganisms and malignant tumor cells (10, 11). Therefore, the treatment of autoimmune diseases is still a difficult problem, and researchers are increasingly interested in the treatment of autoimmune diseases with natural products (12, 13).

*Curcuma longa L.* has been used as a traditional Chinese medicinal material mainly as turmeric for thousands of years in China (14). Curcumin is the most effective ingredient extracted from the rhizomes of ginger plants such as turmeric, curcuma, turmeric, and calamus (15). A number of *in vitro* and *in vivo* experiments showed that curcumin has various pharmacological effects such as regulating immunity, anti-oxidation, inhibiting inflammation, anti-tumor, anti-angiogenesis, anti-coagulation, and scavenging free radicals (15, 16). These studies suggest that curcumin may play a regulatory role by altering the activities of enzymes, receptors, and related transcription factors (17). Numerous randomized controlled trials (RCTs) have shown that curcumin can alleviate many human diseases, including autoimmune diseases, with the main mechanisms in regulating immunity and inhibiting inflammation. Also, curcumin is administered with few side effects, making it a potential alternative to NSAIDs and other drugs with known severe side effects (18–21). In recent years, a large number of RCTs have been published, so it is urgent to summarize and summarize the efficacy and safety of curcumin in autoimmune diseases. This study would provide future clinicians with better evidence for clinical practice by conducting a comprehensive systematic review and meta-analysis of these RCTs, as well as providing more details for future clinical trial design.

## 2 Materials and Methods

### 2.1 Protocol

This systematic review and meta-analysis were conducted strictly in accordance with the protocol (CRD42021289248) and PRISMA-guidelines (see **Supplementary Materials**) (22).

### 2.2 Literature Search Strategy

The English databases (Medline complete, Web of Science, PubMed and Embase) and Chinese databases (Sinomed, Wanfang Database, China National Knowledge Infrastructure, VIP Database) were searched from the establishment of the database to February 2022. We also searched Cochrane Library and The ClinicalTrials.gov. The search strategy of Pubmed and Embase is shown in **Table S1** as an example.

### 2.3.Inclusion and Exclusion Criteria

#### 2.3.1 Participants

The patient was diagnosed with an autoimmune disease by accepted diagnostic criteria.

#### 2.3.2 Intervention

The therapy of the experimental group was curcumin or Curcuma longa Extract preparation, regardless of dosage form, intervention dose, administration route, etc. The therapy of the control group was placebo, conventional therapy, or other curcumin-free therapy.

#### 2.3.3 Outcomes

Outcomes are efficacy indicators, inflammatory indicators and safety indicators.

#### 2.3.4 Design

The study design was RCTs.

#### 2.3.5 Exclusion Criteria

(1) Literature with repeated publications, data and incomplete data. (2) Secondary literature and conference papers such as experience exchange, meta-analysis and review. (3) Animal experiments. (4) The control group therapy contains curcumin or Curcuma longa Extract. (5) abstract only.

### 2.4 Literature Screening and Data Extraction

First, a preliminary search was carried out in Chinese and English databases, after eliminating duplicate literature, systematic reviews, reviews and animal experiments. After further reading the titles and abstracts to eliminate obviously irrelevant literature, according to the inclusion and exclusion criteria, two researchers independently screened eligible literature and extracted data. Data extraction was performed independently by two researchers, and if there was disagreement, they were discussed with other researchers.

### 2.5 Quality Assessment

Quality assessment adopts the RCT risk of bias assessment tool recommended by the Cochrane Handbook (23). Quality assessment was performed independently by two researchers, and if there was disagreement, they were discussed with other researchers.

### 2.6 Statistical Analysis

Data analysis was performed with RevMan 5.3 software provided by the Cochrane Collaboration (24). For continuous variables, mean difference (MD) and 95% confidence interval (CI) were used as effect analysis statistics. The heterogeneity among the results of the included studies was analyzed by the χ 2 test (the test level was α=0.1), and the size of the heterogeneity was quantitatively judged by I2. If there was little or no heterogeneity among study results (P>0.1, I2 ≤ 50%), a fixed-effect model was used. If the heterogeneity among the results of each study was large (P ≤ 0.1, I2>50%), the source of heterogeneity was further analyzed, and the random effect model was used after excluding the influence of obvious clinical heterogeneity (25). The test level of the meta-analysis was set to α=0.05.

## 3 Results

### 3.1 Results of the Search

**Figure 1:**
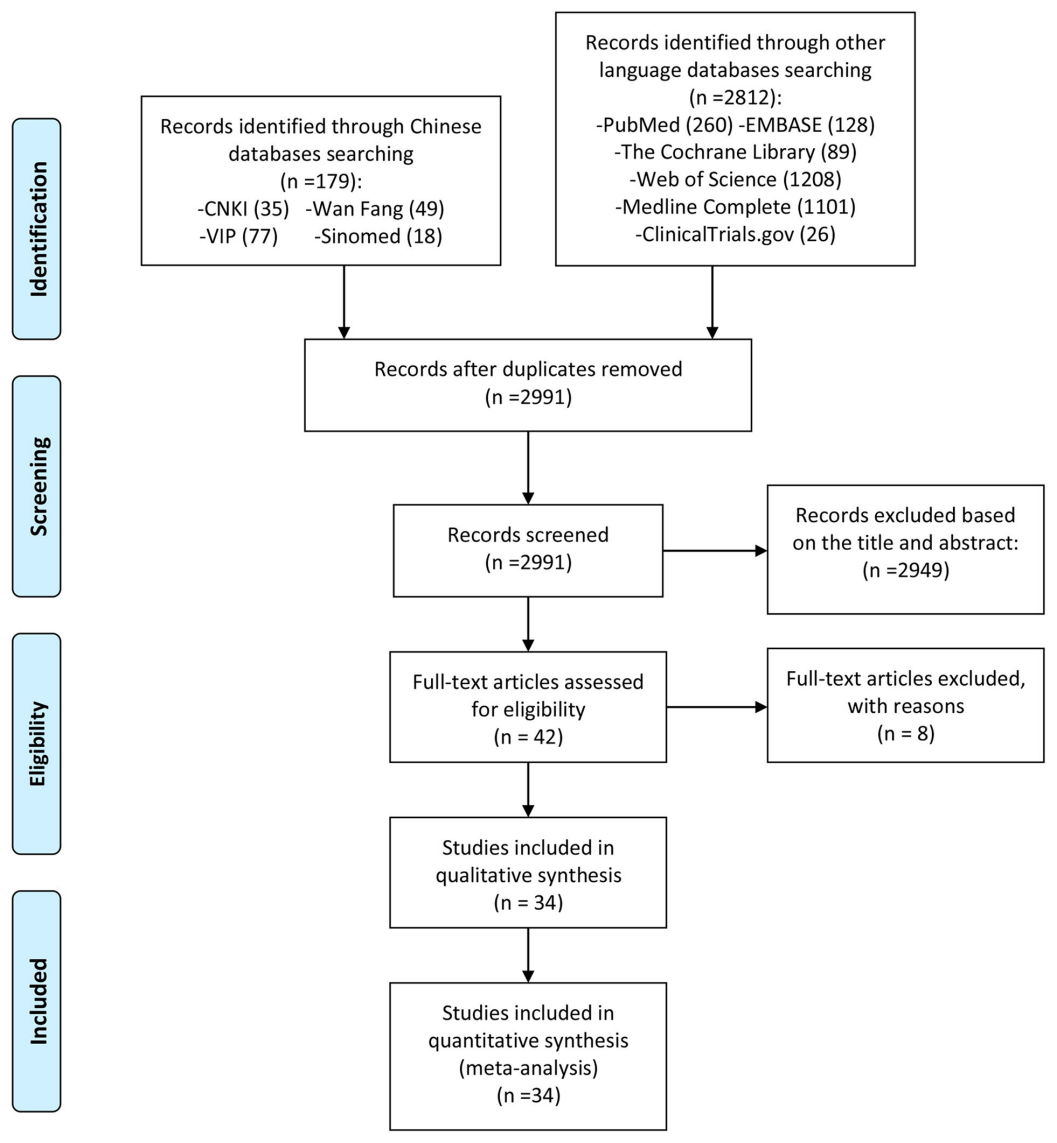
Flow diagram of clinical trials.

A total of 2991 literatures were obtained through computer database search and other methods. After deduplication, after reading the title and abstract, excluding the literature review, review, non-clinical research and obviously irrelevant literature, a total of 42 clinical studies were collected. Read the full text further to exclude articles that did not meet the inclusion criteria (26–33). Finally, a total of 34 RCT studies were included. The process and results of screening literature are shown in **Figure 1**.

### 3.2 Description of Included Trials

A total of 34 records were included, involving 31 RCTs and 10 types of autoimmune disease: AS, BD, Crohn’s Disease, MS, Oral lichen planus, Psoriasis, RA, SLE, Takayasu arteritis, UC. Dolati et al. 2019 (34) and Dolati et al. 2018 (35) belong to the same RCT and thus merge into “Dolati et al. 2019 (34, 35)” Zhu and Zhu 2019 (36) and Zhu 2019 (37) belong to the same RCT and thus merge into “Zhu and Zhu 2019 (36, 37)”; Abbasian et al. 2021 (38) and Farzaneh et al. 2022 (39) belong to the same RCT and thus merge into “Farzaneh et al. 2022 (38, 39)”. Amalraj et al. 2017 (40) and Jacob et al. 2018 (41) used two different doses of curcumin intervention (high-dose group and low-dose group), so they were divided into Amalraj et al. 2017a (low-dose group), Amalraj et al. 2017b (high-dose group), Jacob et al. 2018a (low-dose group), Jacob et al. 2018b (high-dose group). The interventions of Chandran and Goel 2012 (42) were divided into Curcumin 500 mg and Curcumin 500 mg+diclofenac sodium 50 mg, so they were also divided into Chandran et al. 2012a (Curcumin only) and Chandran et al. 2012b (Curcumin+Diclofenac sodium). The details of study characteristics are presented in **Table 1**.

**Table 1:** Characteristic of selected studies.

Disease	Study	Country	Sample size		Intervention		Outcomes	Age (years)		ESR (mm/h)		CRP (mg/L)		Duration
			Trial group	Control group	Trial group	Control group		Trial group	Control group	Trial group	Control group	Trial group	Control group	
Takayasu arteritis	Shao et al. 2017 (43)	China	120	126	Curcumin 300mg	Placebo	Birmingham Vascular Activity Score (BVAS), inflammation indicator	19.1-52.4	18.9-51.2	110 ± 20	120 ± 10	35 ± 5	36 ± 4	4 weeks
Psoriasis	Antiga et al. 2015 (44)	Italy	25	24	Meriva 2000mg (containing curcumin) + topical methylprednisolone aceponate 0.1% ointment	Placebo + topical methylprednisolone aceponate 0.1% ointment	Psoriasis Lesion Area and Severity Index (PASI), adverse events	19-62	22-59	–	–	–	–	12 weeks
	Bilia et al. 2018 (45)	Italy	14	15	Nanocurcumin 3000mg + Acitretin	Placebo + Acitretin	PASI, adverse events	29-59	24-63	–	–	–	–	16 weeks
SLE	Khajehdehi et al. 2012 (46)	Iran	12	12	Turmeric 1500mg	Placebo	Adverse events	32.2 ± 11.4	35 ± 10.4	–	–	–	–	12 weeks
	Singgih et al. 2017 (47)	Indonesia	20	20	Curcumin 60mg + cholecalciferol	Placebo + cholecalciferol	SLEDAI, inflammatory factor	27.9 ± 7.9	30.3 ± 10.0	49.9 ± 9	41.6 ± 23.3	80 ± 50	60 ± 20	12 weeks
MS	Dolati et al. 2019 (34, 35)	Iran	25	25	Nanocurcumin 80mg	Placebo	EDSS, inflammatory factor	35.2 ± 4.2	34.6 ± 5.3	–	–	–	–	24 weeks
	Petracca et al. 2021 (48)	Italy	40	40	BCM 95-micronized curcumin with turmeric essential oils 1000mg + IFN β-1a 44 μg	Placebo + IFN β-1a 44 μg	EDSS, inflammatory factor, adverse events	36.5 ± 8.6	34.3 ± 9.4	–	–	–	–	96 weeks
AS	Ahmadi et al. 2019 (49)	Iran	12	12	Nanocurcumin	Placebo	Inflammatory factor	23-32	27-46	–	–	–	–	16 weeks
BD	Farzaneh et al. 2022 (39)	Iran	17	15	Nanocurcumin 80mg	Placebo	BDCAF, Inflammatory factor, adverse events	41.35 ± 12.42	40.67 ± 8.01	–	–	–	–	8 weeks
UC	Hanai et al. 2006 (50)	Japan	43	39	Curcumin 2000mg + sulfasalazine 1000-3000mg or mesalamine 1500mg-3000mg	Placebo + sulfasalazine 1000-3000mg or mesalamine 1500mg-3000mg	clinical activity index, adverse events	45.2 ± 15.8	39.7 ± 14.2	–	–	–	–	24 weeks
	Singla et al. 2014 (51)	India	14	16	Curcumin enema + 5-Aminosalicylic acid	Placebo enema + 5-Aminosalicylic acid	Clinical response, clinical remission, Endoscopic remission, adverse events	32.7 ± 8.9	35.5 ± 13.8	–	–	–	–	8 weeks
	Lang et al. 2015 (52)	Israel, Hong Kong (China), and Cyprus	25	22	Curcumin 3000mg + mesalamine	Placebo + mesalamine	Clinical response, clinical remission, Endoscopic remission, adverse events	40.4 ± 12.7	41.4 ± 13.9	–	–	–	–	4 weeks
	Kedia et al. 2017 (53)	India	16	25	Curcumin 150mg + mesalamine	Placebo + mesalamine	Clinical response, clinical remission, ESR, Clinical activity index, Endoscopic remission, adverse events	36 ± 12	34 ± 7	3 ± 2	4 ± 3	–	–	8 weeks
	Masoodi et al. 2018 (54)	Iran	28	28	Curcumin 240mg + mesalamine	Placebo + mesalamine	Clinical activity index	38.21 ± 16.37	36.04 ± 11.78	–	–	–	–	4 weeks
	Sadeghi et al. 2020 (55)	Iran	31	32	Curcumin 240mg	Placebo	Clinical response, clinical remission, clinical activity index, ESR, hs-CRP, adverse events	40.1 ± 13.2	40.6 ± 12.4	23.5 ± 16.3	20.6 ± 12.3	3.1 ± 3.2	2.0 ± 2.4	8 weeks
	Banerjee et al. 2021 (56)	India	30	32	Curcumin 100mg + mesalamine	Placebo + mesalamine	Clinical response, clinical remission, Endoscopic remission, adverse events	33.56 ± 10.1	34.66 ± 10.27	–	–	–	–	12 weeks
	Zhu and Zhu 2019 (36, 37)	China	23	23	Curcumin 300mg + Mesalamine	Placebo + Mesalamine	Clinical activity index, Clinical response, clinical remission, Endoscopic remission, ESR, CRP, adverse events	41.43 ± 12.98	42.13 ± 11.07	26.86 ± 7.45	25.34 ± 8.18	15.53 ± 1.76	16.06 ± 1.39	4 weeks
	Wang and Wang 2019 (57, 58)	China	40	40	Turmeric decoction (50g/150mL) enema + Infliximab	Infliximab	Clinical response, clinical remission, Endoscopic remission, CRP	36.8 ± 11.4	37.3 ± 10.9	–	–	47.24 ± 9.34	45.76 ± 7.35	22 weeks
Crohn’s Disease	Bommelaer et al. 2020 (59)	France	26	27	Curcumin 3000mg + azathioprine	Placebo + azathioprine	Postoperative recurrence, adverse events	35.0 ± 10.5	37.6 ± 13.8	–	–	–	–	24 weeks
	Sugimoto et al. 2020 (60)	Japan	17	9	Curcumin 360mg	Placebo	Clinical response, clinical remission, Endoscopic remission, adverse events	36.3 ± 8.9	32.9 ± 13.4	28.8 ± 23.5	42.6 ± 29.9	104 ± 149	149 ± 134	12 weeks
RA	Javadi et al. 2019 (61)	Iran	24	25	Nanocurcumin 120mg	Placebo	DAS28, ESR, adverse events	53.71 ± 2.75	56.28 ± 2.5	23.71 ± 19.9	19.92 ± 14.07	–	–	12 weeks
	Amalraj et al. 2017 (40)	India	24	12	Curcumin 250mg or 500mg	Placebo	DAS28, ESR, CRP, adverse events	36.7 ± 10.7 (250mg); 38.3 ± 5.8 (500mg)	39.6 ± 8.8	175.9 ± 12.9; 181.7 ± 4.8	180.2 ± 12.4	97 ± 15;121 ± 18	97 ± 15	12 weeks
	Jacob et al. 2018 (41)	India	16	8	Curcumin 250mg or 500mg	Placebo	DAS28, ESR, CRP	18-65	18-65	179.12 ± 13.85; 189.37 ± 8.01	183.75 ± 11.97	97 ± 17;114 ± 22	99 ± 16	12 weeks
	Chandran and Goel 2012 (42)	India	30	15	Curcumin 500 mg or Curcumin 500 mg+diclofenac sodium 50 mg	Diclofenac sodium 50 mg	DAS28, ESR, CRP, adverse events	47.8 ± 8.60 (Curcumin only); 47 ± 16.22 (combination)	48.87 ± 10.78	28 ± 23.7; 28.75 ± 20.9	27.08 ± 17.1	5.34 ± 4.12; 9.11 ± 9.93	3.3 ± 2.4	8 weeks
	Pourhabibi-Zarandi et al. 2022 (62)	Iran	22	22	Curcumin 500 mg	placebo	ESR, CRP	50.68 ± 9.93	50.36 ± 9.70	29.09 ± 8.74	24.32 ± 6.99	20.40 ± 14.30	20.34 ± 10.66	8 weeks
Oral lichen planus	Chainani-Wu et al. 2007 (63)	the U.S.	16	12	Curcuminoids 2000mg + Prednisone 60mg	Prednisone 60mg	Modified oral mucositis index, adverse events	60.6 ± 7.5	60.6 ± 9.8	–	–	–	–	7 weeks
	Chainani-Wu et al. 2012 (64)	the U.S.	10	10	Curcuminoids 6000mg	Placebo	Modified oral mucositis index, adverse events	60.8 ± 8.6	56.2 ± 11.7	–	–	20 (10-30)	25 (10-60)	2 weeks
	Kia et al. 2015 (65)	Iran	25	25	5% curcumin paste (ad us est.)	0.1% triamcinolone (ad us est.)	Thongprasom score, adverse events	49.24 ± 8.17	52.08 ± 9.20	–	–	–	–	4 weeks
	Amirchaghmaghi et al. 2016 (66)	Iran	12	8	Curcumin 2000mg +Mouthwash Dexamethasone 0.5 mg	Placebo + Mouthwash Dexamethasone 0.5 mg	Thongprasom score	49.42± 11.22	52.75± 9.43	–	–	–	–	4 weeks
	Thomas et al. 2017 (67)	India	25	25	1% curcumin gel (ad us est.)	0.1% triamcinolone acetonide (ad us est.)	Modified oral mucositis index	20-70		–	–	–	–	12 weeks
	Kia et al. 2020 (68)	Iran	29	28	Nanocurcumin 80mg	Prednisolone 10mg	Lesion size	51.86 ± 9.94	53.67 ± 8.90	–	–	–	–	4 weeks

### 3.3 Risk of Bias of Included Studies

**Figure 2:**
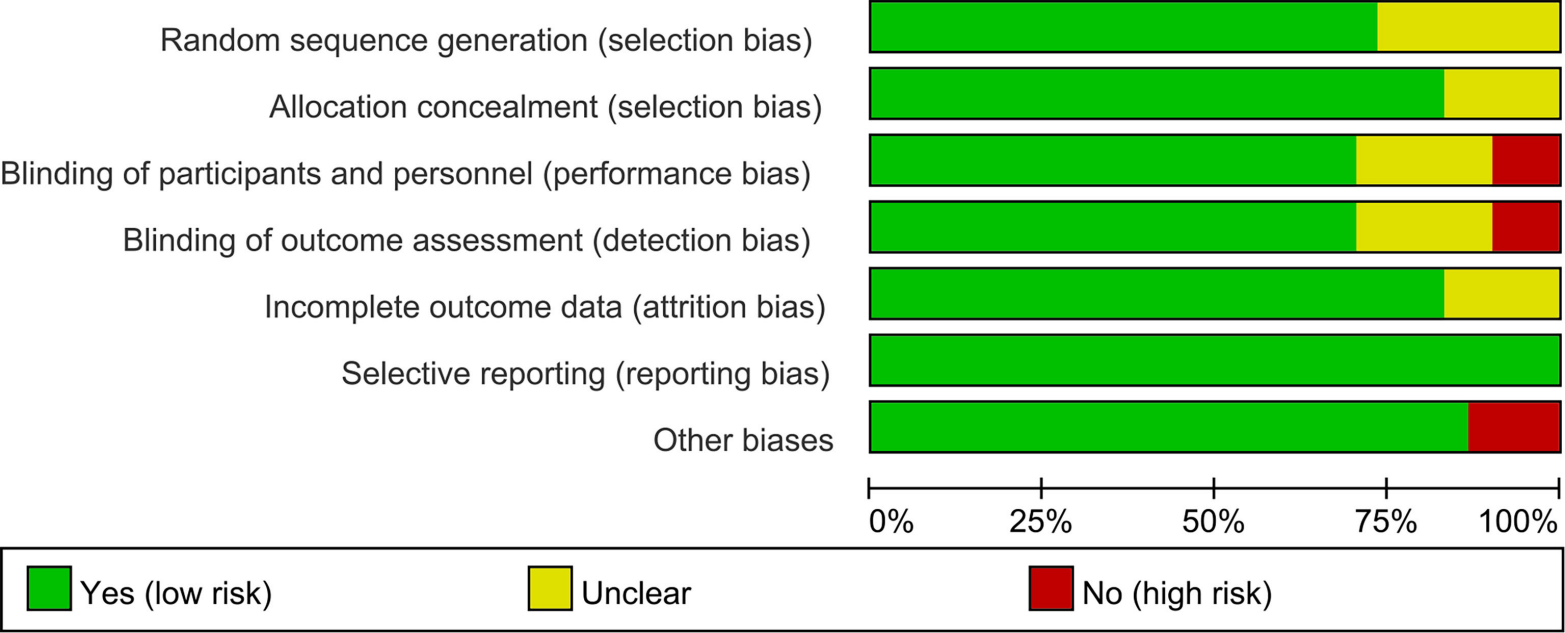
Risk of bias graph.

**Figure 3:**
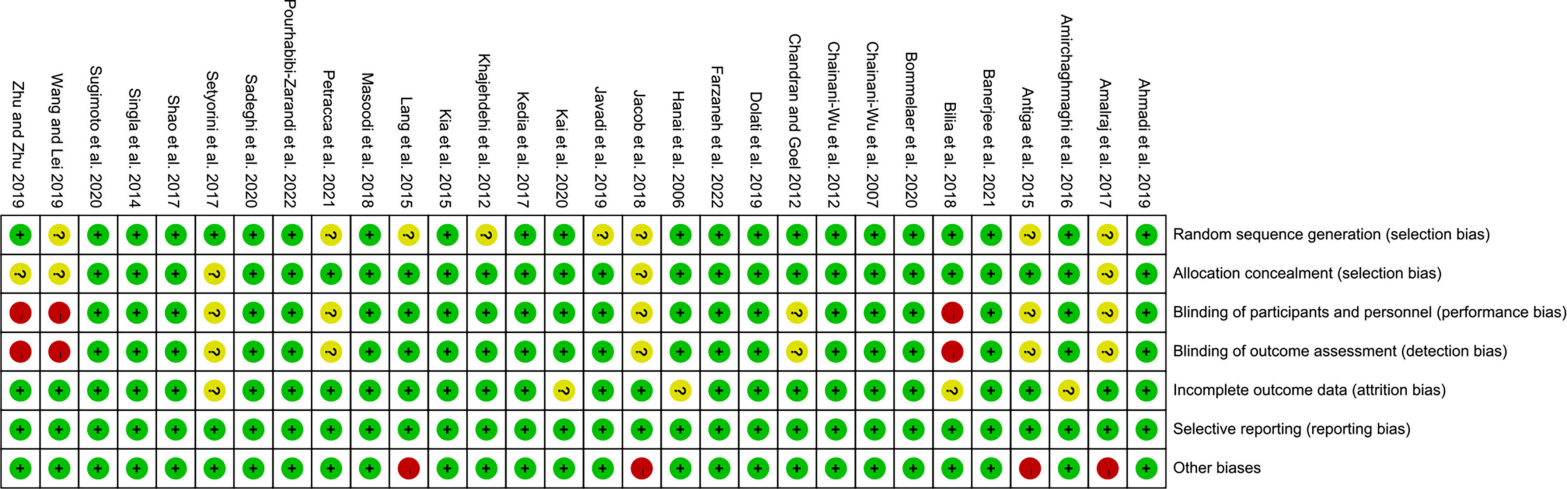
Risk of bias summary.

The summary and graph of risk of bias ware shown in **Figures 2**, **3**.

### 3.4 Curcumin and Curcuma longa Extract in the Treatment of Takayasu Arteritis

Only Shao et al. 2017 (43) reported the effects and safety of curcumin in the treatment of Takayasu arteritis. The study included 246 patients with Takayasu arteritis (120 in the experimental group and 126 in the control group) involving BVAS and inflammation indicators. The results showed that BVAS score after curcumin treatment was lower than that of placebo group, and TNF-α, CRP and ESR were decreased (P<0.05).

### 3.5 Curcumin and Curcuma longa Extract in the Treatment of Psoriasis

Two RCTs reported on the treatment of Psoriasis with Curcumin and Curcuma longa Extract, the data involved can be combined for meta-analysis, and therefore would be described according to the results of the meta-analysis.

#### 3.5.1 PASI

**Figure 4:**
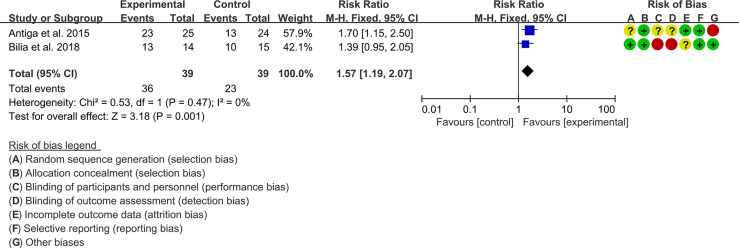
PASI 50.

**Figure 5:**
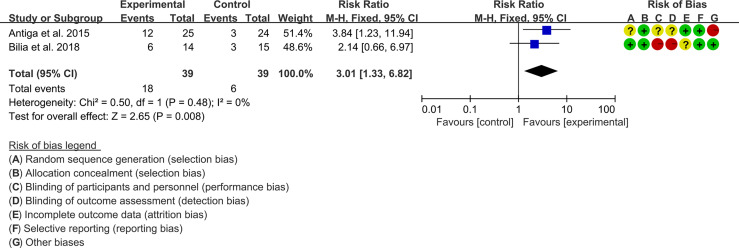
PASI 75.

**Figure 6:**
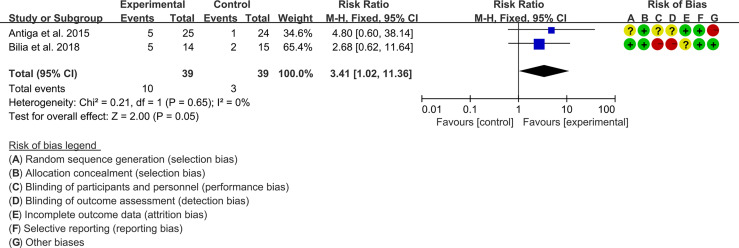
PASI 90.

Two (2) RCTs reported the PASI. The heterogeneity test showed low heterogeneity (PASI50: I2 = 0%, P=0.47; PASI75: I2 = 0%, P=0.48; PASI90: I2 = 0%, P=0.65), so the fixed-effects model was used. The summary results showed that addition of curcumin improved PASI50 (RR=1.57, 95%CI[1.19, 2.07], P=0.001), PASI75 (RR=3.01, 95%CI[1.33, 6.82], P=0.008) and PASI90 (RR=3.41, 95%CI[1.02, 11.36], P=0.05) compared to the control group (**Figures 4**–**6**).

#### 3.5.2 Adverse Events

**Figure 7:**
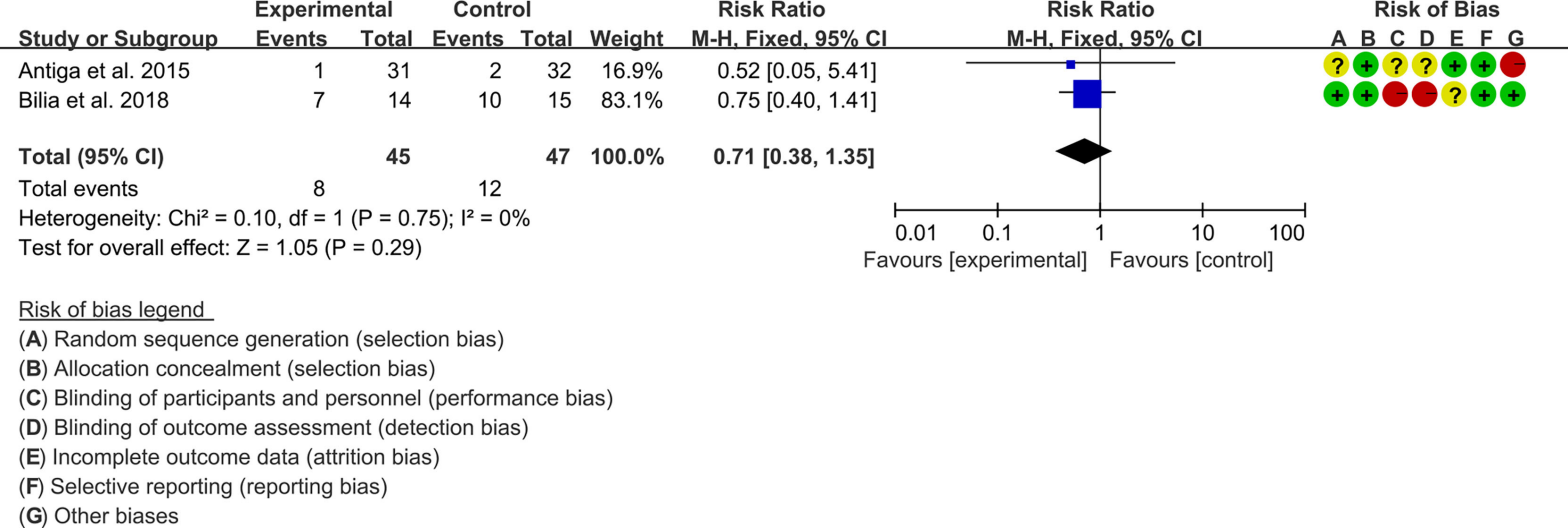
Adverse events.

Two (2) RCTs reported the adverse events. The heterogeneity test showed low heterogeneity (I2 = 0%, P=0.75), so the fixed-effects model was used. The summary results showed that compared with control group, the addition of curcumin did not increase the incidence of adverse events (RR=0.71, 95%CI[0.38, 1.35], P=0.29) (**Figure 7**).

### 3.6 Curcumin and Curcuma longa Extract in the Treatment of SLE

Two (2) RCTs explored the effects and safety of curcumin and curcuma longa Extract in the treatment of SLE. Khajehdehi et al. 2012 (46) included 24 patients with lupus nephritis and no adverse effects related to turmeric supplementation were observed during the trial. Singgih et al. 2017 (47) used Curcumin 60mg combined with cholecalciferol as the treatment regimen for the trial group, with a total of 40 participants. They found that the improvement of SLEDAI, the decrease of IL-6 and the decrease of TGF-β1 by Curcumin 60mg combined with cholecalciferol had no statistical difference compared with the control group (P>0.05).

### 3.7 Curcumin and Curcuma longa Extract in the Treatment of MS

Two (2) RCTs explored the effects and safety of curcumin and curcuma longa Extract in the treatment of MS. Dolati et al. 2019 (34, 35) utilized Nanocurcumin 80mg for patients with MS. They found that compared with placebo controls, curcumin decreased EDSS, increased Treg cells of blood peripheral mononuclear cells, and increased the expression of FoxP3, TGF-β and IL-10 mRNA in blood peripheral mononuclear cells (P<0.05) and also increased serum TGF-β and IL-10 levels (P<0.05). Petracca et al. 2021 (48) used curcumin combined with IFN β-1a therapy to treat patients with MS; they stated that the study dropout rate was too high to draw firm conclusions. Although they found no significant difference in EDSS between the curcumin+IFN β-1a group and placebo+IFN β-1a group (P>0.05), they found that curcumin increased the efficacy of IFN beta-1a on the inflammatory radiological symptoms of MS (P<0.05). They also found that the addition of curcumin did not increase the probability of adverse reactions.

### 3.8 Curcumin and Curcuma longa Extract in the Treatment of AS

Only Ahmadi et al., 2019 explored the effects and safety of curcumin and curcuma longa Extract in the treatment of AS. They included 12 patients who received nanocurcumin and 12 who received a placebo. They found that compared with the control group, Treg cells in the nano-curcumin group increased significantly, the levels of IL-10 and TGF-β increased, and the level of IL-6 decreased (P<0.05).

### 3.9 Curcumin and Curcuma longa Extract in the Treatment of BD

Only Farzaneh et al. 2022 (39) explored the effects and safety of curcumin and curcuma longa Extract in the treatment of BD. They included 17 patients in the nanocurcumin group and 15 in the placebo group. They found that BDCAF was decreased in patients treated with curcumin (P<0.05). They also found that Treg cells, the levels of IL-10 and TGF-β in the nano-curcumin group increased significantly, while IL-17 and IL-23 decreased (P<0.05). No obvious adverse events were observed in the curcumin group and the control group, suggesting that Nanocurcumin is relatively safe.

### 3.10 Curcumin and Curcuma longa Extract in the Treatment of UC

Nine RCTs reported the effects and safety of curcumin and curcuma longa Extract in the treatment of UC. The data involved can be combined for meta-analysis, and therefore would be described according to the results of the meta-analysis.

#### 3.10.1 Clinical Activity Index

**Figure 8:**
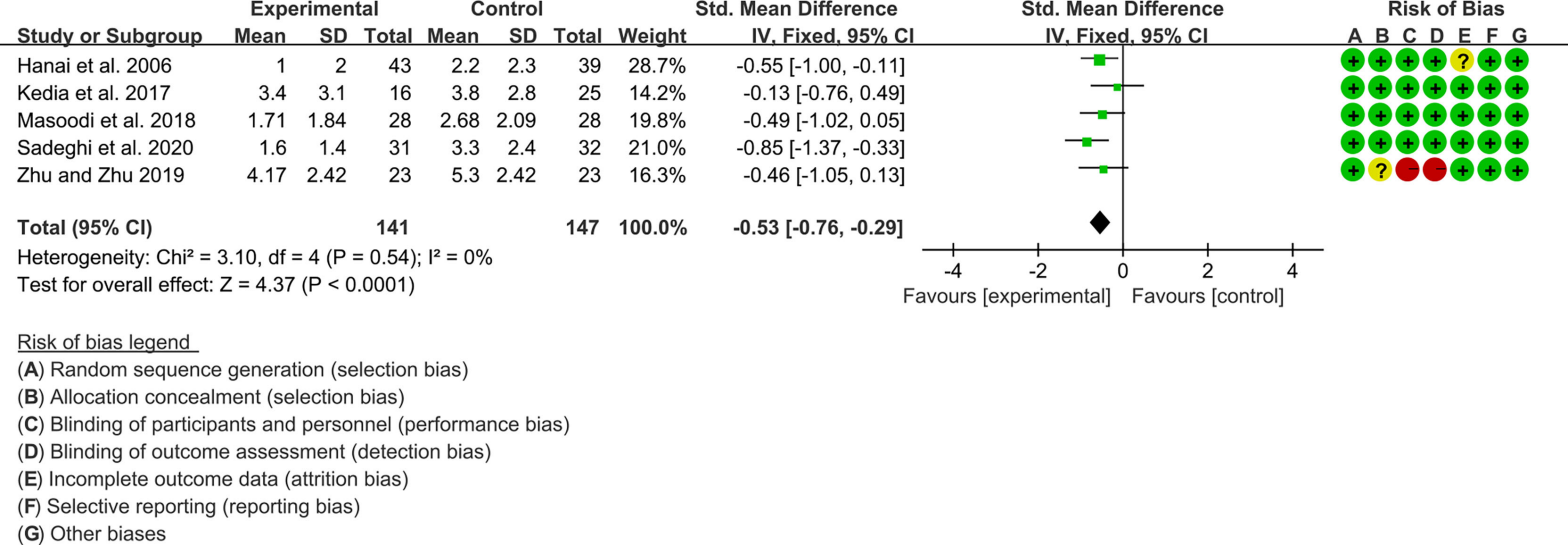
Clinical activity index.

Five (5) RCTs reported clinical activity index. Due to the different scales used, SMD was used to represent the effect size. The heterogeneity test showed low heterogeneity (I2 = 0%, P=0.54), so the fixed-effects model was used. The summary results showed that compared with control group, the addition of curcumin decreased the clinical activity index (SMD=-0.53, 95%CI[-0.76, -0.29], P<0.0001) (**Figure 8**).

#### 3.10.2 Clinical Efficacy

**Figure 9:**
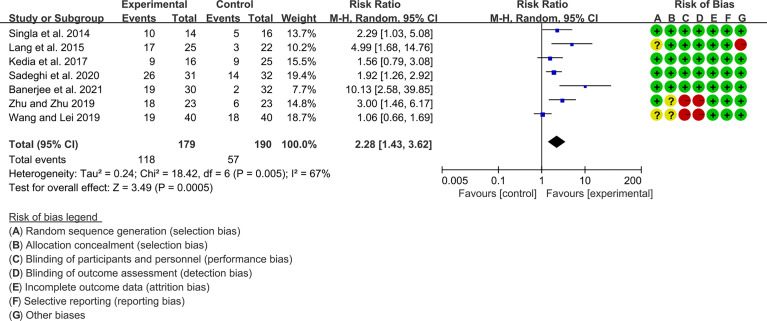
Clinical remission.

**Figure 10:**
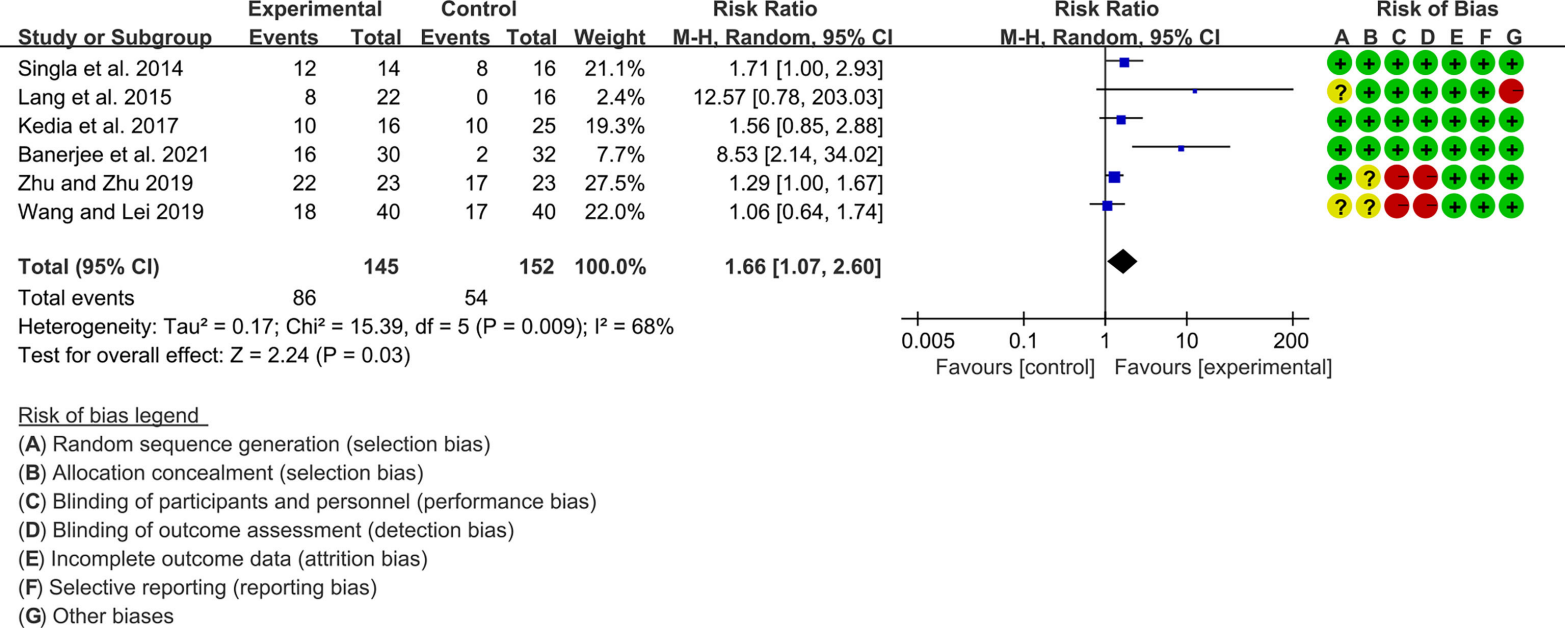
Endoscopic remission.

**Figure 11:**
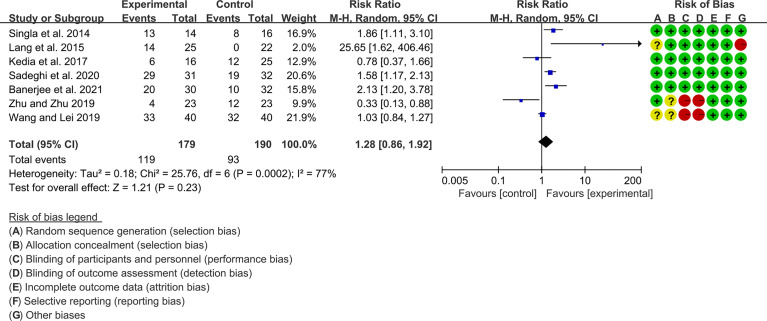
Clinical response.

Seven (7) RCTs reported clinical remission and clinical response rats, and 6 reported endoscopic remission rates. The heterogeneity test showed high heterogeneity (clinical remission: I2 = 67%, P=0.005; clinical response: I2 = 77%, P=0.0002; endoscopic remission: I2 = 68%, P=0.009.), so the random-effects model was used. The summary results showed that compared with control group, the addition of curcumin increased the clinical remission rates (RR=2.28, 95%CI[1.43, 3.62], P=0.0005) and endoscopic remission rates (RR=1.66, 95%CI[1.07, 2.60], P=0.03) (**Figures 9**, **10**), while the addition of curcumin did not increase the clinical remission rates (RR=1.28, 95%CI[0.86, 1.92], P=0.23) (**Figure 11**)

#### 3.10.3 Inflammatory Factor

**Figure 12:**
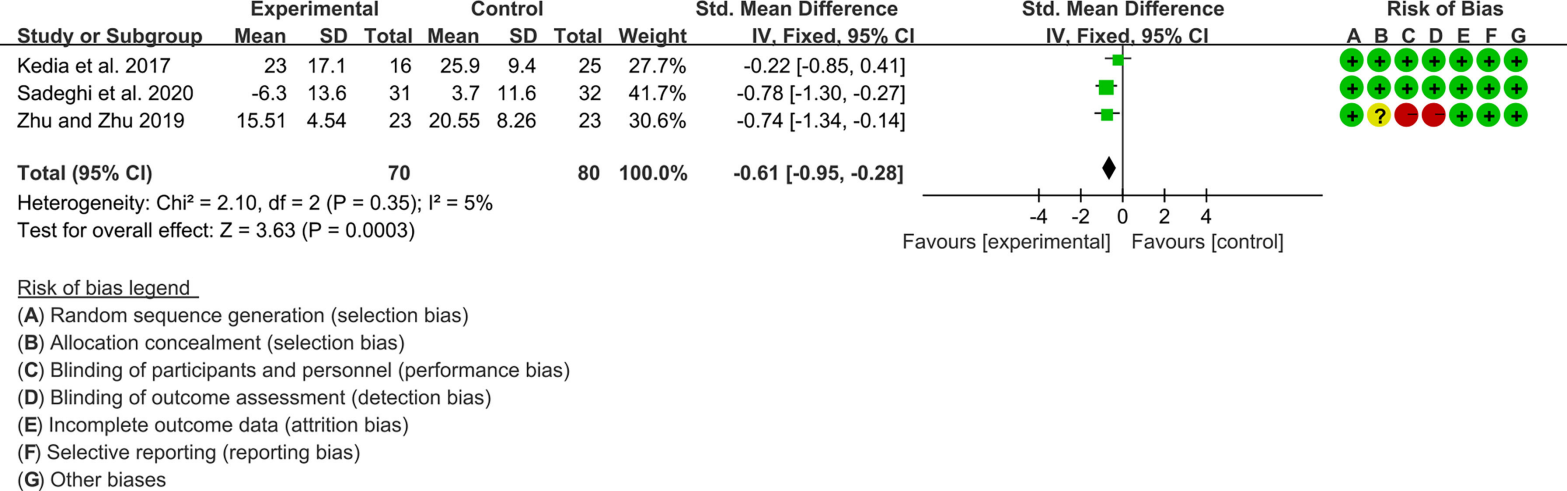
ESR.

Three (3) RCTs reported ESR and CRP. For ESR, the heterogeneity test showed low heterogeneity (I2 = 5%, P=0.35), so the fixed-effects model was used. The summary results showed that compared with control group, the addition of curcumin decreased the ESR (SMD=-0.61, 95%CI[-0.95, -0.28], P=0.0003) (**Figure 12**).

**Figure 13:**
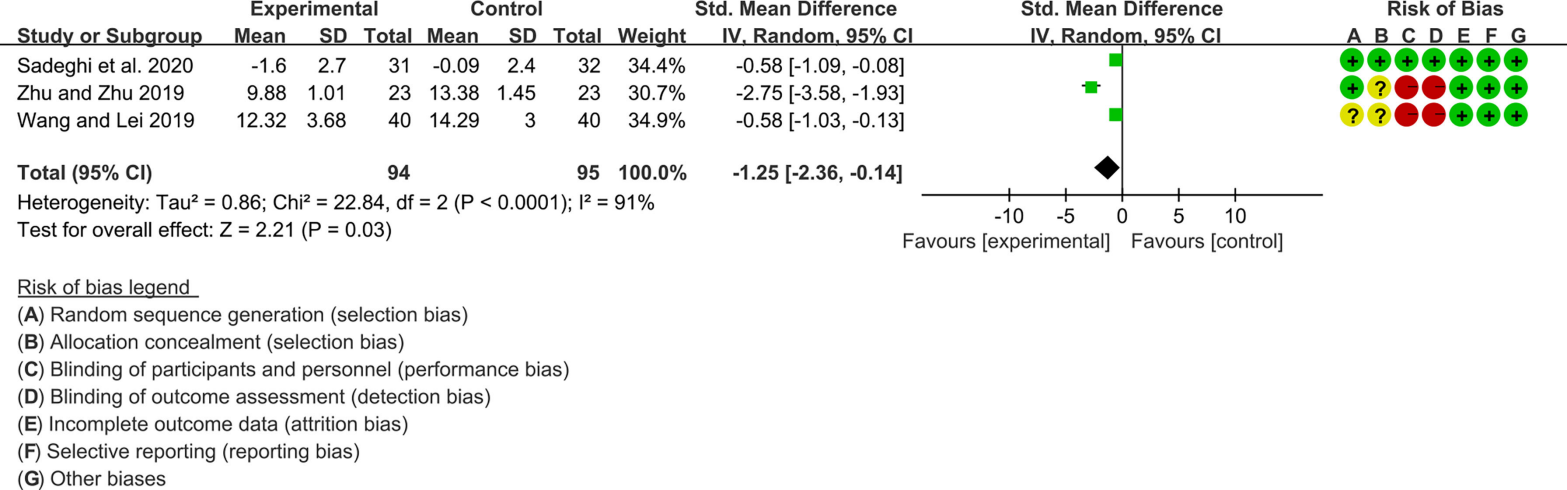
CRP.

For CRP, the heterogeneity test showed high heterogeneity (I2 = 91%, P<0.0001), so the random-effects model was used. The summary results showed that compared with control group, the addition of curcumin decreased the CRP (SMD=-1.25, 95%CI[-2.36, -0.14], P=0.03) (**Figure 13**)

#### 3.10.4 Adverse Events

**Figure 14:**
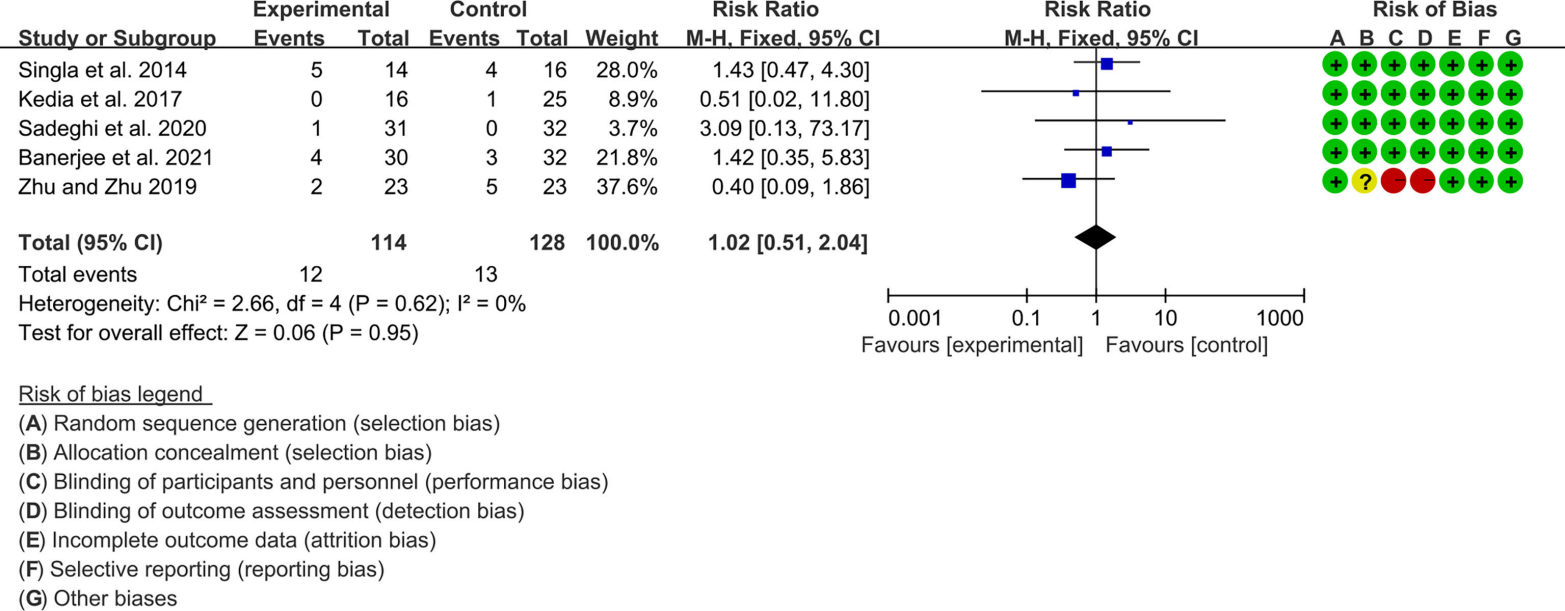
Adverse events.

Five (5) RCTs reported the adverse events. The heterogeneity test showed low heterogeneity (I2 = 0%, P=0.62), so the fixed-effects model was used. The summary results showed that compared with control group, the addition of curcumin did not increase the incidence of adverse events (RR=1.02, 95%CI[0.51, 2.03], P=0.95) (**Figure 14**).

### 3.11 Curcumin and Curcuma longa Extract in the Treatment of Crohn’s Disease

Two (2) RCTs explored the effects and safety of curcumin and curcuma longa Extract in the treatment of Crohn’s Disease. Bommelaer et al. 2020 (59) found that Curcumin was no more effective than placebo in preventing Crohn’s disease relapse and probably did not improve patients’ quality of life. Thiopurine was discontinued due to nausea and abdominal pain in 2 patients each in the curcumin and placebo groups. In addition, adverse events were reported in 2 patients in the placebo group (1 pregnancy and 1 abscess) and 5 patients in the curcumin group (2 abdominal pain, 1 vomiting, 1 migraine, and 1 cervical squamous cell carcinoma); and these are not considered to be related to the addition of curcumin. However, (60) found that with the addition of curcumin, clinical remission and Endoscopic remission improved, and no serious adverse events were observed. Only one patient showed mild appetite loss, which did not affect the completion of the clinical trial.

### 3.12 Curcumin and Curcuma longa Extract in the Treatment of RA

Five RCTs reported on the treatment of RA with Curcumin and Curcuma longa Extract, the data involved can be combined for meta-analysis, and therefore would be described according to the results of the meta-analysis.

#### 3.12.1 DAS28

**Figure 15:**
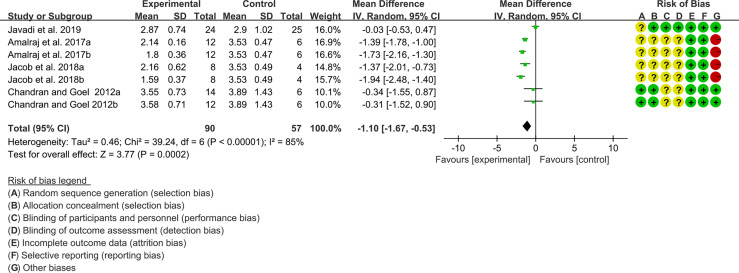
DAS28.

Four (4) RCTs reported the DAS28. The heterogeneity test showed high heterogeneity (I2 = 85%, P<0.00001), so the random-effects model was used. The summary results showed that compared with control group, the addition of curcumin decreased the DAS28 (WMD=-1.10, 95%CI[-1.67, -0.53], P=0.0002) (**Figure 15**).

#### 3.12.2 Inflammatory Factor

**Figure 16:**
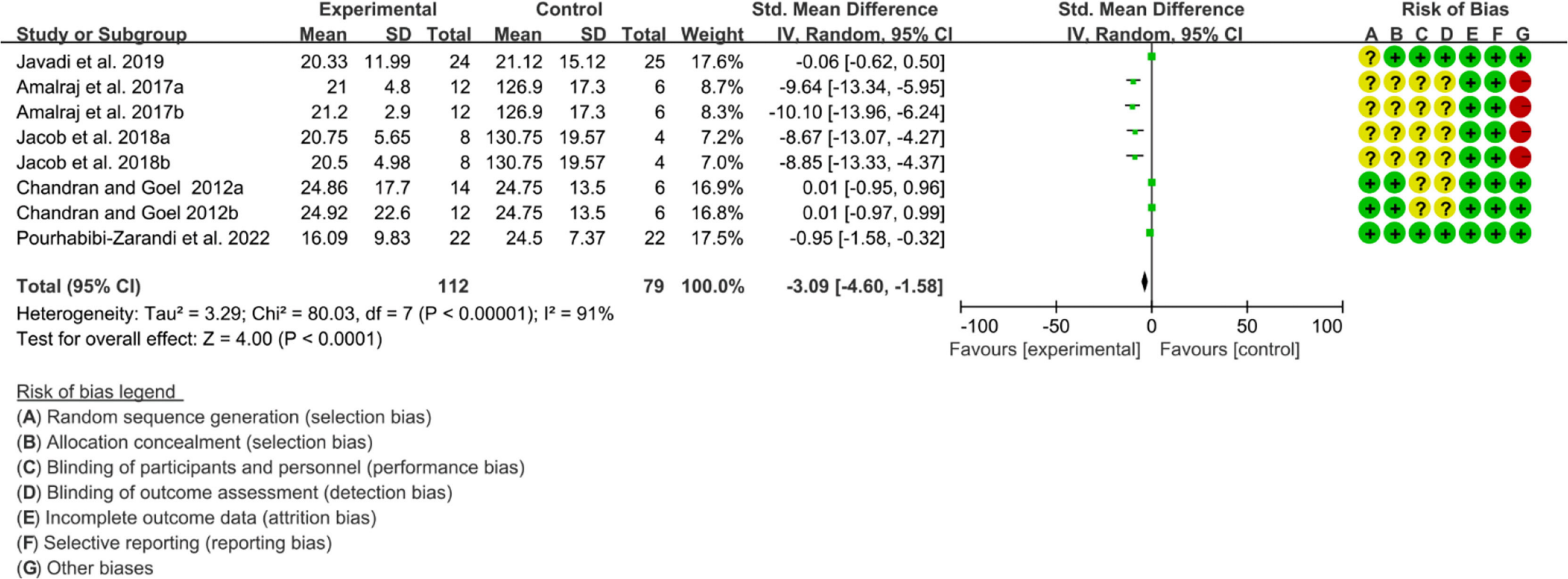
ESR.

**Figure 17:**
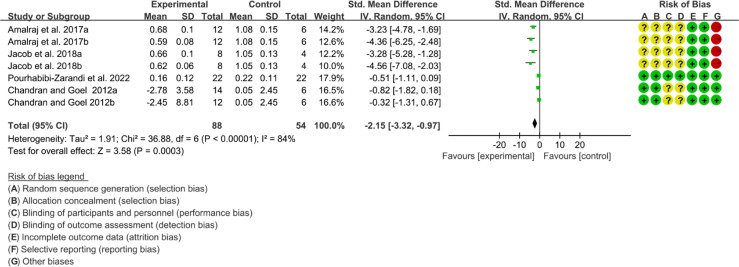
CRP.

Five (5) RCTs reported the ESR. The heterogeneity test showed high heterogeneity (I2 = 91%, P<0.00001), so the random-effects model was used. The summary results showed that compared with control group, the addition of curcumin decreased the ESR (SMD=-3.09, 95%CI[-4.60, -1.58], P<0.0001) (**Figure 16**). Four (4) RCTs reported the CRP. The heterogeneity test showed high heterogeneity (I2 = 91%, P<0.00001), so the random-effects model was used. The summary results showed that compared with control group, the addition of curcumin decreased the CRP (SMD=-2.15, 95%CI[-3.32, -0.97], P=0.0003) (**Figure 17**).

#### 3.12.3 Adverse Events

**Figure 18:**
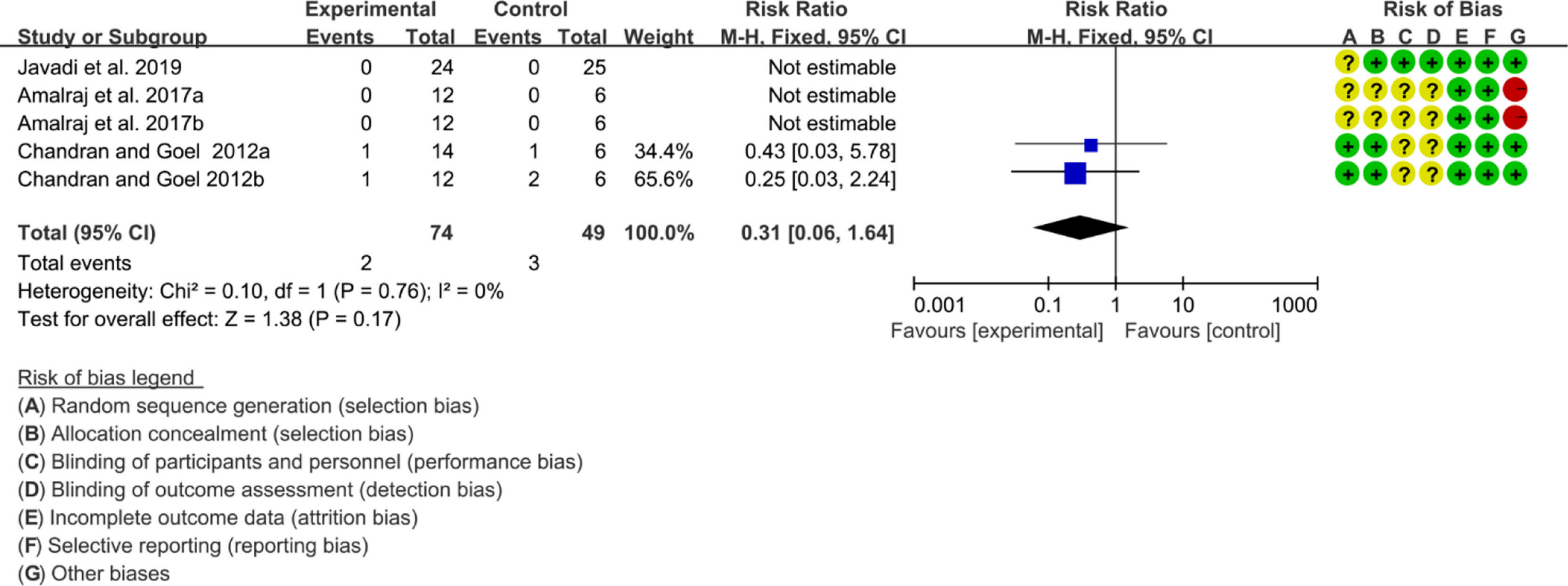
Adverse events.

Three (3) RCTs reported the adverse events. The heterogeneity test showed low heterogeneity (I2 = 0%, P=0.78), so the fixed-effects model was used. The summary results showed that compared with control group, the addition of curcumin did not increase the incidence of adverse events (RR=0.31, 95%CI[0.06, 1.64], P=0.17) (**Figure 18**).

### 3.13 Curcumin and Curcuma longa Extract in the Treatment of Oral lichen planus

**Figure 19:**
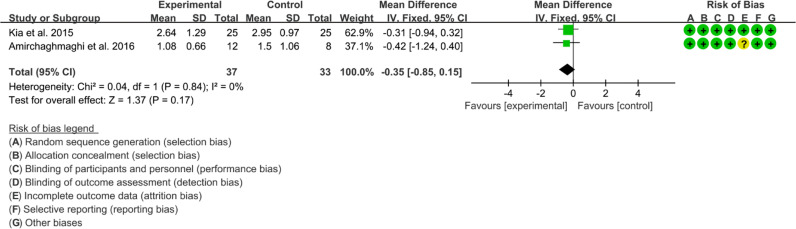
Thongprasom score.

Six RCTs reported Curcumin and Curcuma longa Extract in the Treatment of Oral lichen planus. (64, 67) found that Curcumin may decrease Modified oral mucositis index (P<0.05). However, (68) found no significant difference in efficacy between Curcumin and Prednisolone. Since their indicators could not be combined, Meta-analysis was not conducted. (63–65) found no significant adverse events. (65, 66) reported Thongprasom score, which could be combined. The heterogeneity test showed low heterogeneity (I2 = 0%, P=0.78), so the fixed-effects model was used. The summary results showed that compared with control group, the addition of curcumin did not decrease Thongprasom score (WMD=-0.35, 95%CI[-0.85, 0.15], P=0.17) (**Figure 19**).

## 4 Discussion

### 4.1 Mechanisms of Curcumin Regulation of Immune Cells in Autoimmune Diseases

Autoimmune diseases are a series of diseases caused by the immune system’s immune response to self-antigens, leading to self-tissue damage or dysfunction (69, 70). Many autoimmune diseases are characterized by the production of autoantibodies that bind to the host’s own proteins or form immune complexes that deposit in tissues (71, 72). Any organ may become a target organ of autoimmunity, including skin, joints, kidneys, blood vessels, etc. (73). Inflammatory effects caused by autoantibodies are mediated by binding to Fc receptors on leukocytes and are an important cause of downstream tissue damage. Meanwhile, autoantibodies can also directly mediate tissue damage in disease through complement activation (74). During the developmental stage of the disease, genetic and environmental factors may in turn interact to promote the development of autoimmunity, ultimately leading to tissue inflammation and damage (75).

In autoimmune diseases, the balance between the body’s recognition of foreign pathogens and the immune system’s attack on self-antigens is disrupted, resulting in abnormal immune tolerance. During the breakdown of immune tolerance, initiating tissues provide a microenvironment that influences immune cell differentiation leading to the activation of adaptive immunity. Type 1 interferons produced by innate immune cells play a central role in systemic autoimmunity and activate B and T cells, while B cell-derived autoantibodies stimulate dendritic cells, which in turn produce type 1 interferons. Therefore, the adaptive immune response pathway and the innate immune response pathway play important roles in the pathogenesis of systemic autoimmunity (76–78). Studies have shown that systemic autoimmune diseases (such as RA, SLE, etc.) share common genetic risk loci, and the pathogenic mechanisms may be similar (79–84). Dysregulation of basic innate immune strategies, such as the complement pathway, IFN synthesis and response to IFN, and neuromodulatory mechanisms of immune responses, contribute to autoimmunity and tissue damage in SLE (85–88).

Adaptive immune cells in the adaptive immune system also play an important role. With the activation of dendritic cells, more interferon is produced in the autoimmune response, which promotes and maintains the self-reaction, keeps the activated T cells and B cells in a vicious circle, and produces autoantibodies. Various cytokines activate Naive T cells to promote their (Naive T cells) differentiation into helper T cells (Th cells) and Regulatory T (Treg) through different transcription factors. These Th cells and Treg further secrete different cytokines, which in turn recruit different immune cells and coordinate different immune effector mechanisms (89, 90). There are also interactions between different subgroups, forming complex regulation and action mechanisms (91).

Curcumin has been shown to be a potent immunomodulator, which can modulate the activity of T cells, B cells, macrophages, neutrophils, NK cells, and dendritic cells (92, 93). The diverse pharmacological activities of curcumin stem from its ability to interact with different biological targets and signaling pathways (94). The immunomodulatory activity of curcumin may involve the direct targeting (activation) of TLRs (such as TLR4: the receptor for LPS) through pathogen-associated molecular patterns (PAMPs) (95). It may also be attributed to the regulation of various transcription factors, such as nuclear factor (NF-κB), activator protein 1 (AP-1), signal transducer and activator of transcription (STAT) and their downstream signaling pathways (96–100).

In the regulation of immune cells of the innate immune system: curcumin inhibits the maturation of DCs by inhibiting IDO expression through a cyclooxygenase (COX-2)/prostaglandin E2 (PG-E2)-dependent pathway (101, 102). Several curcumins can reduce neutrophil recruitment to inflamed tissues through direct effects on neutrophil chemotaxis and chemotaxis (103, 104). Curcumin can reduce the production of antibodies (IgG2a, IgE and IgG1, especially IgG1) in rat splenocytes in response to LPS (105, 106). The immunomodulatory effects of curcumin on CD8+ and CD4+ T cell subsets such as curcumin can inhibit the production of Th1 cytokine profile in CD4+ T cells by inhibiting the production of IL-12 in macrophages (107). From a morphological point of view, intestinal dendritic cells (DC) treated with curcumin caused intestinal T cells to become unresponsive. Furthermore, the antigen-presenting properties of curcumin-treated DCs are blocked, resulting in reduced induction of the adaptive immune system (108). DCs treated with curcumin stimulated the differentiation of intestinal Tregs, which prevented disease progression in an animal model of colitis (109). Other studies have shown that curcumin pretreatment of DCs inhibits LPS-stimulated NF-κB p65 translocation and MAPK phosphorylation, thereby reducing inflammation (99). Curcumin also reduced proinflammatory cytokine upregulation, primarily IL-12, and hindered Th1-type responses in DCs. In addition, curcumin has been shown to reduce the expression of ICAM-1 (intercellular adhesion molecule-1) and CD11c, proteins involved in cell adhesion and T cell stimulation, possibly through an AP-1-dependent pathway.

In modulating B-cell immunity: *In vitro* studies have also shown that curcumin reduces serum levels of BAFF following total IgG, Toll-like receptor (TLR) 4 stimulation. It also reduced B cell activation in MRL/lpr mice and reduced lupus nephritis in MRL/lpr mice. This may represent a holistic treatment strategy in the management of SLE (110).

In regulating T cell immunity: Low-dose curcumin treatment (0.1 and 1 μg/ml) was also shown to reduce Th17 cells, IL-17A levels, and increase Treg and TGF-β1 concentrations on CD4+ T cells in SLE patients compared with normal controls (111). Various studies have reported that curcumin can precisely alter Th17/Treg balance on CD4+ T cells and alleviate organ damage in SLE subjects (111). Kalim et al. also found reduction in Th17 and improvement in Treg cells following administration of 200 mg kg day-1 curcumin in a mouse model of SLE (112). Furthermore, curcumin intervention in T cells obtained from a mouse model of psoriasis showed that 10 μM curcumin concentration significantly blocked the secretion of IL-17, IL-22, IFN-γ, IL-2, IL-8 and TNF-α (113). Xie etal. (114) found a reduction in the severity of EAE in rats treated with curcumin. Curcumin attenuated the severity of experimental allergic encephalomyelitis (EAE) due to inhibition of Th17 cell differentiation and growth by downregulating IL-6, IL-21, RORΔt signaling and STAT3 phosphorylation. Along these lines, Liu et al. found that the viability of CD4+ T cells *in vitro* was considerably inhibited by curcumin treatment, and furthermore, curcumin induced significant apoptosis of Th1, Th17 and Treg cells (115).

### 4.2 Curcumin for Takayasu Arteritis

Only one RCT reported the effects and safety of curcumin in the treatment of Takayasu arteritis. They found that curcumin may improve BVAS and inflammation indicators. Therefore, TNF-α was considered to be significantly associated with a decrease in BVAS score (γ2 = 0.81, P = 0.016), ESR (γ2 = 0.76, P = 0.037), and plasma CRP level (γ2 = 0.79, p = 0.041). The findings suggest that curcumin may significantly improve the therapeutic effect through its anti-TNF-α effect (43). However, more reports are needed to further verify the therapeutic effect and safety of curcumin on Takayasu arteritis.

### 4.3 Curcumin for Psoriasis

This study included 2 relevant RCTs and found that curcumin improved PASI in patients with psoriasis without increasing side effects compared with controls. Psoriasis is a multifactorial mediated chronic inflammatory hereditary skin disease characterized by inflammatory erythema overlying multiple layers of silvery-white mica-like scales with localized or widespread systemic distribution (116). The incidence of psoriasis vulgaris in the general population is relatively high, and the incidence in European and American countries is about 2.1%, while that in China is about 0.123%, which can seriously affect the interpersonal communication and daily life of patients (117). At present, it is generally believed that psoriasis is caused by immune imbalance caused by external stimuli such as infection, trauma, mental stress and other factors under the influence of the patient’s genetic susceptibility (118). The role of immunity in the pathogenesis of psoriasis has been widely accepted, and psoriasis genome-wide scans have also shown that most susceptibility genes are associated with immunity (119, 120). The immunological mechanism of psoriasis is complex. The main participants are keratinocytes and a variety of immune cells, including dendritic cells, macrophages, neutrophils and T cells in innate and adaptive immunity (121, 122). The relationship between innate immunity and adaptive immune system of psoriasis is realized by cytokines. The representative inflammatory factors include TNF-α, IFN-γ, IL-17 A and IL-1, and the inhibitory inflammatory factors (regulatory inflammatory factors) include IL-4, IL-10 and TGF-β (123, 124). Activated cytokines bind to corresponding receptors as specific ligands, and coordinately mediate the pathological changes of psoriasis. Both the adaptive immune system and the innate immune system can mediate the production of inflammatory mediators, and the latter play an important role in the induction and maintenance of dermal and epidermal psoriasis pathological changes (125–127). Different topical and systemic treatment options are currently available for the treatment of psoriasis, but they have suboptimal clinical outcomes and risk side effects (128, 129). Multiple current studies have reported that curcumin can reduce oxidative stress in psoriatic lesions (130). In addition, the therapeutic efficacy of curcumin may also be related to its ability to inhibit phosphorylase kinase. Curcumin may inhibit the proliferation of psoriasis-like cells by down-regulating pro-inflammatory cell (HaCaT cell) factors such as IL-17, TNF-α, INF-γ and IL-6 (131). Furthermore, curcumin significantly enhanced skin barrier function by upregulating involucrin (iNV) and filaggrin (FLG) (58, 132). Curcumin can inhibit the pro-proliferation effect of VEGF on HaCaT cells and promote the apoptosis of HaCaT cells. *In vivo* studies have shown that curcumin has no observed side effects in the treatment of psoriasis patients (26, 133), and the U.S. Food and Drug Administration (FDA) has defined curcumin as “generally regarded as safe” (GRAS). In summary, curcumin has great potential as a treatment for psoriasis. However, more RCTs are needed to further verify the therapeutic effect and safety of curcumin on Psoriasis.

### 4.4 Curcumin for SLE

Only Two (2) RCTs explored the effects and safety of curcumin and curcuma longa Extract in the treatment of SLE. They found no increase in side effects with the addition of curcumin, and found that curcumin may improve SLEDAI and reduce IL-6 and TGF-β1. However, since the data could not be combined for meta-analysis, they need to be interpreted with caution. SLE is the most diverse autoimmune disease, which can affect any organ of the body, can have a wide range of clinical and immunological manifestations, and its incidence is increasing year by year (134). SLE is characterized by the presence of multiple autoantibodies in serum (135). Autoantibodies closely related to SLE mainly include antinuclear antibodies, anti-Sm antibodies, anti-double-stranded DNA antibodies, and anti-SSA antibodies (136). High titers of double-stranded DNA are associated with SLE glomerular deposition and nephritis activity, and antiphospholipid antibodies are associated with SLE prone to coagulation (137). Treatment options for SLE depend on the organ involved. Patients with mild symptoms can be treated with low-dose corticosteroids and are usually controlled, but moderate and severe disease may require higher doses of corticosteroids or other immunosuppressants (138). Pharmacological studies of curcumin on SLE showed that SLE is characterized by the activation of the complement system, and curcumin can inhibit the complement cascade (139). Curcumin can prevent oxidative stress of oxidatively modified proteins by binding to proteins, and can also inhibit the activation of B cells to effectively treat SLE (140). Low doses of curcumin can also specifically regulate Th17/Treg balance in CD4 + T cell cultures from SLE (111). Curcumin reduced proteinuria and serum levels of IgG1, IgG2a and anti-dsDNA IgG antibodies in female NZB/W F1 mice (141). In summary, curcumin may enhance regulatory responses involving T reg cells by inhibiting antibody-antigen interactions, reducing autoantigen-autoantibody deposition in tissues and various microvascular beds, and inhibiting antibody production. However, more RCTs are needed to further verify the therapeutic effect and safety of curcumin on SLE.

### 4.5 Curcumin for MS

Only Two (2) RCTs explored the effects and safety of curcumin and curcuma longa Extract in the treatment of MS. They both found that curcumin could reduce inflammatory factors, however, their findings were different on the improvement of EDSS. However, since the data could not be combined for meta-analysis, they need to be interpreted with caution. MS is an inflammatory demyelinating disease of the central nervous system that affects millions of people around the world, and approximately 30% of MS patients develop clinical paralysis (142). Destruction of myelinating oligodendrocytes is a pathological feature of MS, and axonal deformation leads to irreversible long-term disability (143). The activation of immune cells, the secretion of inflammatory cytokines and the differentiation of Th1 cells are the key processes in the pathogenesis of MS (144). EAE is an autoimmune disease of the central nervous system that has been used as a model to study the pathogenesis of MS and to test the efficacy of treatments for MS (145, 146). (147) observed the protective effect of curcumin in the treatment of EAE in SJL/J rats, and found that 50-100 mg of curcumin administered by gavage every other day could alleviate the symptoms of EAE in SJL/J rats. In addition, curcumin can inhibit neural antigen-induced T cell proliferation, Th1 differentiation, and TNFγ production (148). Yang etal. (149) found that curcumin can significantly improve the clinical symptoms of EAE rats, inhibit the infiltration of inflammatory cells, and promote the recovery of rats, thereby treating MS. In EAE-induced rats, curcumin treatment was found to significantly reduce the number of inflammatory cells infiltrating the spinal cord (114). Curcumin treatment was also associated with upregulation of IL-10 levels and increased percentage of CD4+CD25+-Foxp3+ Treg cells in the CNS and lymphoid organs in EAE-induced C57BL/6 mice. Furthermore, curcumin ameliorated EAE in SJL/J mice by inhibiting IL-12 signaling through the JAK-STAT pathway, resulting in reduced Th1 differentiation (150). *In vivo* treatment with curcumin has been shown to increase the expression of PPARγ in the central nervous system and lymphoid organs of EAE mice, suggesting that it is involved in the regulation of Th1/Th17 responses in EAE (151). Curcumin may inhibit IL-17 mRNA expression and T cell INF-γ levels in MS patients (150). Curcumin may inhibit the proliferation of TEM cells and the production of pro-inflammatory cytokines by inhibiting the hKv1.3 channel, which contributes to the curcumin’s efficacy in the treatment of autoimmune diseases (152). These basic experimental results all suggest that curcumin may be used to treat MS, therefore, more RCTs are still needed to further verify the therapeutic effect and safety of curcumin on MS.

### 4.6 Curcumin for AS

Only (49) explored the effects and safety of curcumin and curcuma longa Extract in the treatment of AS. They found that Treg cells increased significantly, the levels of IL-10 and TGF-β increased, and the level of IL-6 decreased after curcumin intervention. AS is a disease characterized by inflammation of the sacroiliac joints and spinal attachments as the main symptom, mainly affecting the axial skeleton, sacroiliac joints, and peripheral joints, and can cause structural changes and dysfunction (153, 154). The main clinical features of AS are intermittent low back pain, discomfort and stiffness in other parts of the body (155), which eventually progresses to spinal immobility and rigidity (156). Recent studies have shown that AS is also a T lymphocyte-mediated disease, which is supported by changes in the frequency of CD4 + T cells in the peripheral blood of patients with AS, including an increase in Th17 frequency with Th2 and a decrease in CD4 + CD25 + Treg (157–159). The results of most studies suggest that the Treg/Th17 ratio is reduced in the PB of AS patients, and speculate that altered immunophenotypes may play a role in the pathogenesis of these diseases, thus modulating the balance between Treg and Th17 may reduce disease activity (160, 161). Recent studies have found that curcumin may enhance Treg differentiation by increasing FoxP3 expression (111, 162), which is consistent with the RCT findings of Ahmadi et al., 2019. However, more RCTs are still needed to further verify the therapeutic effect and safety of curcumin on MS.

### 4.7 Curcumin for BD

Only (39) explored the effects and safety of curcumin and curcuma longa Extract in the treatment of BD. They found that BDCAF was decreased in patients treated with curcumin. They also found that Treg cells, the levels of IL-10 and TGF-β in the nanocurcumin group increased significantly, while IL-17 and IL-23 decreased. No obvious adverse events were observed in the curcumin group and the control group. BD is a systemic inflammatory disease with oral and vulvar ulcers and uveitis as the main clinical manifestations (163). Pathological manifestations are vasculitis involving various vascular diameters, with a tendency to thrombosis (164). The pathogenesis of BD is unclear. In addition to environmental and genetic factors, abnormal T cell immune responses are also involved in the pathogenesis, involving innate immune γδ T cells, identification of characteristic antigens, antigen presentation, and adaptive immunity represented by CD4+ and CD8+ T cells. (165, 166). The imbalance of T cell homeostasis is mainly manifested by the activation, proliferation and Treg cell damage of Th1 and Th17 helper T cells (167). The above-mentioned abnormal immune response induces and maintains the pro-inflammatory environment of BD (168). Previous studies have described that curcumin enhances the mucosal healing process of oral ulcers in humans and animals (169–171). Mohammad et al. found that the mRNA expression of IL-1β and the protein production of IL-6 and TNFα were significantly down-regulated in M1 macrophages from BD patients after curcumin (30 µg/ml) intervention (172). Curcumin also reduced macrophage polarization and production of proinflammatory cytokines IL-1β and TNFα in the human macrophage cell line M1 (173–175), which is consistent with the RCT report of (39). However, more RCTs are still needed to further verify the therapeutic effect and safety of curcumin on BD.

### 4.8 Curcumin for UC and Crohn’s Disease

Nine RCTs reported the effects and safety of curcumin and curcuma longa Extract in the treatment of UC. Two (2) RCTs explored the effects and safety of curcumin and curcuma longa Extract in the treatment of Crohn’s Disease. Curcumin may improve the clinical activity index, clinical response and endoscopic response, and decrease the ESR and CRP for patients with UC. (59) found that Curcumin was no more effective than placebo in preventing Crohn’s disease relapse and probably did not improve patients’ quality of life. (60) found that with the addition of curcumin, clinical remission and Endoscopic remission improved. Meanwhile, the addition of curcumin may not increase the incidence of adverse events. However, more RCTs are still needed to further verify the therapeutic effect and safety of curcumin on UC and Crohn’s Disease.

Inflammatory bowel disease (IBD) is currently considered to be an autoimmune disease, mainly including ulcerative colitis (UC) and Crohn’s disease, which are histopathologically manifested as intestinal mucosal inflammation (176, 177). IBD is an autoimmune disease involving autoantibodies and autoreactive CD4+ T lymphocytes. CD4+ T cells are essential for regulating immune homeostasis in the gut, and innate immune abnormalities will cause CD4+ T cells (Th1, Th2, Th17) to produce a large number of pro-inflammatory factors, making the immune response beyond the range of Treg regulation, thereby causing IBD (178, 179). Although the pathogenesis of UC and Crohn’s disease is characterized by an immune response to gut (bacterial) antigens, they differ in the type of inflammatory response. The pathogenesis of Crohn’s disease is mainly related to the inflammatory response dominated by cytokines IL-1, IL-6, IL-8, TNF-α and IFN-γ secreted by Th1 and Th17 cells (180). UC is related to the inflammatory response dominated by cytokines such as IL-4, IL-5, IL-9 and 1L-13 secreted by Th2 cells (181). Therefore, it is possible to try to regulate the CD4+ T cell population and related cytokines and signaling pathways as one of the means of IBD treatment (182, 183). Zhang etal. (148) found that curcumin decreased the expression of Th1 cytokines and up-regulated the expression of Th2 cytokines in the colonic mucosa. Lee et al. (179) also found that curcumin could alleviate ovalbumin (OVA)-induced food allergy by regulating the balance between Th1/Th2. This indicates that curcumin plays an anti-inflammatory role by changing the immune balance between Th1-Th2 to Th2-type immunity. Li etal. (184) used curcumin to treat dextran sulfate sodium (DSS)-induced IBD, and found that p38MAPK protein and mRNA expression were significantly reduced, and TNF-α production was also reduced. This suggests that curcumin may play a role in the treatment of IBD by inhibiting the p38MAPK pathway and reducing the production of TNF-α. In addition, curcumin may reduce apoptosis and alleviate colon injury by regulating the p38MAPK and JNK pathways (185). Curcumin can exert an anti-inflammatory effect in experimental colitis by inhibiting the STAT3 pathway (186). Yang etal. (187) showed that curcumin can inhibit the expression of cyclinD1 and CDK4 with cell proliferation effect by inhibiting the STAT-3 signaling pathway, showing anti-inflammatory effect in IBD. Guo etal. (188) found that flavin may increase the level of peroxisome proliferator-activated receptor-g (PPAR-g), inhibit the STAT3 signaling pathway, reduce the release of COX-2, and reduce the infiltration of neutrophils.

### 4.9 Curcumin for RA

Five RCTs reported the effects and safety of curcumin and curcuma longa Extract in the treatment of RA. They found that curcumin may decrease DAS28, ESR and CRP. Meanwhile, the addition of curcumin may not increase the incidence of adverse events. However, more RCTs are still needed to further verify the therapeutic effect and safety of curcumin on RA.

RA is a systemic autoimmune disease characterized by chronic destructive joint disease (189, 190), and 0.5% to 1% of the world’s population is affected by RA (191). RA is characterized by synovitis and pannus production, and activated macrophages and dendritic cells are important sources of key inflammatory cytokines, including TNF-α and IL-1, as well as promoting inflammatory cells. Accumulated and synthesized cytokines, such as chemokines, matrix metalloproteinases (MMPs), COX-2 and other inflammatory mediators (192, 193). CD4+ Th cells play a key role in the occurrence of immune responses, B cells contribute to the progression of inflammation, and RA occurs by activating T cells (194). The main drugs for the treatment of RA include NSAIDs, disease-modifying anti-rheumatic drugs (DMARDs), and biological agents (190, 195), but these drugs have serious side effects. Curcumin was found to have anti-rheumatic activity in humans (196). Curcumin can inhibit the occurrence and development of RA mainly by inhibiting inflammatory cytokines and MMPs and blocking signaling pathways, including mitogen-activated protein kinase, activator protein-1 and nuclear factor-κB receptor activator ligand, etc. (197). Shang (198) found that curcumin can effectively inhibit angiogenesis by inhibiting the expression of hypoxia-inducible factor 1-α, thereby protecting the synovium of joints. In addition, curcumin protects chondrocytes by inhibiting the activation of caspase-3 by preventing the integration of IL-1β (199). Curcumin can reduce the gene expression of adhesion molecules, β3 and β7 integrins, thereby reducing joint inflammation in RA (200).

### 4.10 Curcumin for Oral Lichen Planus

Six RCTs reported Curcumin and Curcuma longa Extract in the Treatment of Oral lichen planus. (64), (67) found that curcumin may decrease Modified oral mucositis index. However, (68) found no significant difference in efficacy between Curcumin and Prednisolone. Since their indicators could not be combined, Meta-analysis was not conducted. and meta-analysis showed that curcumin may not reduce Thongprasom score. Therefore, more RCTs are still needed to further verify or modify the therapeutic effect and safety of curcumin on Oral lichen planus. Oral lichen planus is a chronic inflammatory disease of the oral mucosa of unknown etiology (201). At present, its pathogenesis is often related to mental factors, immune factors, infectious factors, endocrine factors, lack of trace elements, microcirculation disorders, and some systemic diseases (202, 203). The basis of Oral lichen planus may be T cell-mediated responses to unknown triggers (204, 205). Observational studies have also shown that patients with Oral lichen planus are more prone to an antioxidant-oxidative stress imbalance (206, 207). Treatment for Oral lichen planus is aimed at managing symptoms. Topical or systemic application of glucocorticoids and immunosuppressants is the main clinical treatment for Oral lichen planus. Although it can obtain a relatively reliable curative effect, its long application period and many adverse reactions lead to poor patient compliance, which is the main problem that plagues clinical diagnosis and treatment (202, 208). Meanwhile, there is still a lack of safer and more effective treatment options for some patients with intractable damage or those with complex systemic history. Curcumin, an active phytochemical in turmeric, is a strongly hydrophobic compound with poor bioavailability, which poses challenges for oral ingestion (209). On the other hand, curcumin was not shown to be toxic at doses of 8 g per day, making it a potentially attractive alternative to drugs currently used to treat various diseases (210).

### 4.11 Implications for the Future

Current pharmacological studies have shown that Curcumin and Curcuma longa Extract seems to reverse some clinical symptoms of many autoimmune diseases by regulating immune inflammatory biological modules, such as inflammatory factors (such as IL-1, IL-17, IL-6, IL-12, TNF-α and IFN-γ) -mediated inflammatory signaling pathways and immune inflammatory cell activation, differentiation and immune function regulation. Curcumin and Curcuma longa Extract is an effective natural compound with a variety of therapeutic pharmacological properties and almost no side effects. Recent studies have shown that curcumin can synergistically enhance the synergistic effect of glucocorticoids and alleviate glucocorticoid-induced osteoporosis (211, 212). Therefore, the future design of RCTs may consider combining Curcumin and Curcuma longa Extract with existing conventional drug therapies such as glucocorticoids. It may be a potential side effect of reducing glucocorticoid dose and preventing glucocorticoid use to autoimmune diseases. Future RCTs should accurately determine the appropriate dose, dosage regimen, treatment duration, and further clarify the mechanism of action related to systemic autoimmune diseases. In terms of treatment and preparation, an important issue in the treatment of autoimmune diseases by oral Curcumin and Curcuma longa Extract is to improve its bioavailability (213). Although a large number of studies have been conducted on curcumin preparations to improve bioavailability, it still needs to be further improved.

## 5 Conclusion

This systematic review and meta-analysis evaluated the efficacy and safety of Curcumin and Curcuma longa Extract in autoimmune diseases, such as AS, BD, Crohn’s Disease, MS, Oral lichen planus, Psoriasis, RA, SLE, Takayasu arteritis, UC. Because of its good clinical safety, the dose of curcumin in the treatment of autoimmune diseases is mainly between 80 mg and 6000 mg. The results of Meta-analysis showed that Curcumin and Curcuma longa Extract had good clinical efficacy in the treatment of Psoriasis, UC and RA, so Curcumin and Curcuma longa Extract could be used in the treatment of the above diseases in the future. The results of Meta-analysis showed that Curcumin and Curcuma longa Extract did not show efficacy in the treatment of oral lichen planus, while Takayasu arteritis, SLE, MS, AS, BD and CD did not report sufficient clinical data for meta-analysis; hence, more RCTs are still needed in the future. Curcumin and Curcuma longa Extract may regulate inflammatory cells (such as Treg) and inflammatory factors (such as CRP, ESR, TNF-α, TGF-β1, IL-6 level), which may be its mechanism for treating autoimmune diseases. However, due to the low quality and small number of RCTs in most autoimmune diseases, the conclusions need to be carefully interpreted. At present, large-sample, multi-center clinical trials are still needed for revision or validation.

## Data Availability Statement

The original contributions presented in the study are included in the article/**Supplementary Material**. Further inquiries can be directed to the corresponding authors.

## Author Contributions

LZ, TY, KY, HC are responsible for the study concept and design. LZ, TY, KY, GY, JL, WX, and HC are responsible for the data collection, data analysis and interpretation; LZ and KY drafted the paper; HC supervised the study; all authors participated in the analysis and interpretation of data and approved the final paper.

## Conflict of Interest

The authors declare that the research was conducted in the absence of any commercial or financial relationships that could be construed as a potential conflict of interest.

## Publisher’s Note

All claims expressed in this article are solely those of the authors and do not necessarily represent those of their affiliated organizations, or those of the publisher, the editors and the reviewers. Any product that may be evaluated in this article, or claim that may be made by its manufacturer, is not guaranteed or endorsed by the publisher.
